# Glycolysis Define Two Prognostic Subgroups of Lung Adenocarcinoma With Different Mutation Characteristics and Immune Infiltration Signatures

**DOI:** 10.3389/fcell.2021.645482

**Published:** 2021-07-22

**Authors:** Chen Huo, Meng-Yu Zhang, Rui Li, Ting-Ting Liu, Jian-Ping Li, Yi-Qing Qu

**Affiliations:** ^1^Department of Pulmonary and Critical Care Medicine, Qilu Hospital, Cheeloo College of Medicine, Shandong University, Shandong Key Laboratory of Infectious Respiratory Diseases, Jinan, China; ^2^Department of Pulmonary and Critical Care Medicine, Qilu Hospital of Shandong University, Shandong Key Laboratory of Infectious Respiratory Diseases, Jinan, China

**Keywords:** lung adenocarcinoma, glycolysis, tumor mutational burdens, prognosis, tumor-infiltrating immune cell

## Abstract

Increasing studies have proved that malignant tumors are associated with energy metabolism. This study was aimed to explore biological variables that impact the prognosis of patients in the glycolysis-related subgroups of lung adenocarcinoma (LUAD). The mRNA expression profiling and mutation data in large LUAD samples were collected from the Cancer Genome Atlas (TCGA) database. Then, we identified the expression level and prognostic value of glycolysis-related genes, as well as the fractions of 22 immune cells in the tumor microenvironment. The differences between glycolysis activity, mutation, and immune infiltrates were discussed in these groups, respectively. Two hundred fifty-five glycolysis-related genes were identified from gene set enrichment analysis (GSEA), of which 43 genes had prognostic values (*p* < 0.05). Next, we constructed a glycolysis-related competing endogenous RNA (ceRNA) network which related to the survival of LUAD. Then, two subgroups of LUAD (clusters 1 and 2) were identified by applying unsupervised consensus clustering to 43 glycolysis-related genes. The survival analysis showed that the cluster 1 patients had a worse prognosis (*p* < 0.001), and upregulated differentially expressed genes (DEGs) are interestingly enriched in malignancy-related biological processes. The differences between the two subgroups are SPTA1, KEAP1, USH2A, and KRAS among top 10 mutated signatures, which may be the underlying mechanism of grouping. Combined high tumor mutational burden (TMB) with tumor subgroups preferably predicts the prognosis of LUAD patients. The CIBERSORT algorithm results revealed that low TMB samples were concerned with increased infiltration level of memory resting CD4+ T cell (*p* = 0.03), resting mast cells (*p* = 0.044), and neutrophils (*p* = 0.002) in cluster 1 and high TMB samples were concerned with increased infiltration level of memory B cells, plasma cells, CD4 memory-activated T cells, macrophages M1, and activated mast cells in cluster 2, while reduced infiltration of monocytes, resting dendritic cells, and resting mast cells was captured in cluster 2. In conclusion, significant different gene expression characteristics were pooled according to the two subgroups of LUAD. The combination of subgroups, TMB and tumor-infiltrating immune cell signature, might be a novel prognostic biomarker in LUAD.

## Introduction

Lung adenocarcinoma (LUAD) is a highly fatal cancer of the respiratory system worldwide, and the 5-year survival rate of LUAD is less than 17% (Hirsch et al., 2017). It is reported that application of biomarkers may provide effective prognostic values in LUAD. For example, CAV1 and DCN participate in regulating LUAD progression (Yan et al., 2019). Despite progress in molecular researches, more prognostic biomarkers need to be further explored in LUAD.

Glycolysis is one of the well-studied pathways of glucose metabolism. Normal mammalian cells are inhibited by glycolysis under aerobic conditions, while malignant tumor cells are active in glycolysis even under a sufficient-oxygen environment. This metabolic feature of aerobic glycolysis is called the Warburg effect, which is manifested by high glucose uptake rate, active glycolysis, and high lactic acid content of metabolites (Allard et al., 1994). Previous studies have confirmed that increased levels of glycolysis are involved in malignancy progression, such as proliferation, invasion, and migration (Ganapathy-Kanniappan and Geschwind, 2013; Jin et al., 2017). It is reported that downregulation of Barx2 promotes aerobic glycolysis and predicts a poor prognosis in non-small cell lung carcinoma (NSCLC) (Chen et al., 2018). Given that, understanding the mechanisms associated with glycolysis could be a major breakthrough for finding potential prognostic targets. In this study, we aimed to explore new biomarker strategies of glycolysis-related tumor subgroups, which was associated with tumor-infiltrating immune cells, a tumor mutational burden (TMB) in LUAD.

The immune system plays key roles in the surveillance and elimination of tumor cells. The malignant phenotype of tumors is determined not only by the internal activity of tumor cells but also by the tumor-infiltrating immune cells in the tumor microenvironment (Swann and Smyth, 2007), which can promote or suppress the development and growth of tumors (Shiao et al., 2011). Previous studies have shown that the infiltration by immune cells in tumor tissues may have a prognostic value (Fridman et al., 2012). Therefore, the signature of tumor-infiltrating immune cells may be regarded as a potential predictive prognostic biomarker.

Tumor cell mutations could change the function and expression of proteins, resulting in the appearance of tumor-specific neoantigens. T-cells then recognize these neoantigens, causing an antitumor response. Increasing the activation of immune cells through immunotherapy may remove immune-mediated tumor cells and ultimately improve the patients’ prognosis. Researchers have identified that high levels of TMB may be more responsive to immunotherapy (Rooney et al., 2015; Fancello et al., 2019), and KRAS mutation can be regarded as a predictive biomarker in lung cancer (Hainsworth and Anthony Greco, 2016). Thus, further studies of mutation signature should be explored to identify prognostic biomarkers for LUAD.

One single prediction method always makes the results inaccurate. Therefore, in the present study, the combination of TMB and tumor-infiltrating immune cells to assess prognosis in different tumor subgroups of LUAD could provide more accurate and effective biomarkers.

## Materials and Methods

### Data Collection, Processing, and Validation

We firstly gathered RNA-sequencing data and prognostic information of LUAD from the GDC tool^1^. Then, we downloaded somatic mutation data of all patients processed by VarScan software, and the “maftools” package and “pheatmap” package in R were used to further analyze the mutation data. Subsequently, the “ConsensusClusterPlus” package was performed to construct consensus analysis and PCA was applied to verify the accuracy of the classification. Eventually, the “limma” package was performed to identify differentially expressed lncRNAs (DElncRNAs), differentially expressed miRNAs (DEmiRNAs), and differentially expressed genes (DEGs) with |logFC > 1| and *p* < 0.05. The independent cohort GSE72094 was downloaded from the Gene Expression Omnibus (GEO) database^2^ for data validation. The dataset contains both expression levels of related genes and clinical information such as age, gender, survival status, and survival time.

### Gene Set Enrichment Analysis (GSEA)

Gene set enrichment analysis^3^ (Subramanian et al., 2005) was used to explore whether the selected gene sets revealed significant differences between the tumors tissues and normal tissues. We obtained glycolysis-related data (KEGG GLYCOLYSIS GLUCONEOGENESIS, BIOCARTA GLYCOLYSIS PATHWAY, HALLMARK GLYCOLYSIS, and REACTOME GLYCOLYSIS) from the MsigDB database^4^. *p* < 0.05 was the standard to assess whether to further investigate.

### Construction of a Glycolysis-Related Competing Endogenous RNA (ceRNA) Network

The miRTarBase^5^ (Chou et al., 2018) and miRDB^6^ (Chen and Wang, 2020) databases were used to obtain miRNA-targeted glycolysis-related genes. StarBase^7^ (Li et al., 2014) was performed to predict relationships between lncRNAs and miRNAs.

### Functional Enrichment Analysis of DEGs and Protein–Protein Interaction (PPI) Network Construction Across Two Clusters

To figure out the functional enrichment of glycolysis-related DEGs, the “GOplot” package and “ClusterProfiler” package were used to further analyze by gene ontology (GO) and Kyoto Encyclopedia of Genes and Genomes (KEGG) pathways, and *p* < 0.05 was considered as statistically significant. Then, a protein–protein interaction (PPI) network was constructed using the STRING database (Szklarczyk et al., 2015) and the cytoHubba plugin of Cytoscape software (Shannon et al., 2003).

### Evaluation of Tumor-Infiltrating Immune Cells

CIBERSORT is a new algorithm used for calculating fractions of 22 immune cells subsets *via* RNA-seq or microarray data. The immune cells included seven types of T cells and three types of B cells, NK cells and various myeloid cells. We selected the samples with *p* < 0.05 to elevate the accuracy.

### Validation of 43 Significant Glycolysis-Related DEGs Using Quantitative Reverse Transcription-Polymerase Chain Reaction (qRT-PCR)

Total RNA was extracted from 16HBE and H1299 cells using the TRIzol reagent (Invitrogen, Carlsbad, CA, United States). After the purity and concentration of the total RNA were determined, the total RNA was reverse transcribed into cDNA using the PrimeScript RT reagent kit (Accurate Biology). The qRT-PCR was performed using the SYBR Green Premix Ex Taq II (Accurate Biology). The PCR conditions were set as follows: 95°C for 30 s, followed by 40 cycles at 95°C for 5 s and 60°C for 30 s for each specific primer. Finally, the relative mRNA expression levels of 43 genes were calculated using the 2^–ΔΔCT^ method. The primer sequences are listed in Supplementary Data Sheet 2.

### Statistical Analysis

Statistical analysis was utilized by R software (version 4.0.3) and GraphPad Prism (version 7.0). The differences of expression levels between two groups were determined by Student’s *t*-test. We used the “survival” packages to process the survival analysis, and survival curves were visualized by the Kaplan–Meier plotter^8^, which was examined by the log-rank test. The difference of infiltrating immune cells between the high-TMB group and the low-TMB group was determined by unpaired *t*-test, as well as the expression level of the glycolysis-related genes between cluster 1 and cluster 2. Differences were considered significant at *p* < 0.05.

## Results

### Glycolysis Is Associated With the Tumorigenesis of LUAD

**FIGURE 1:**
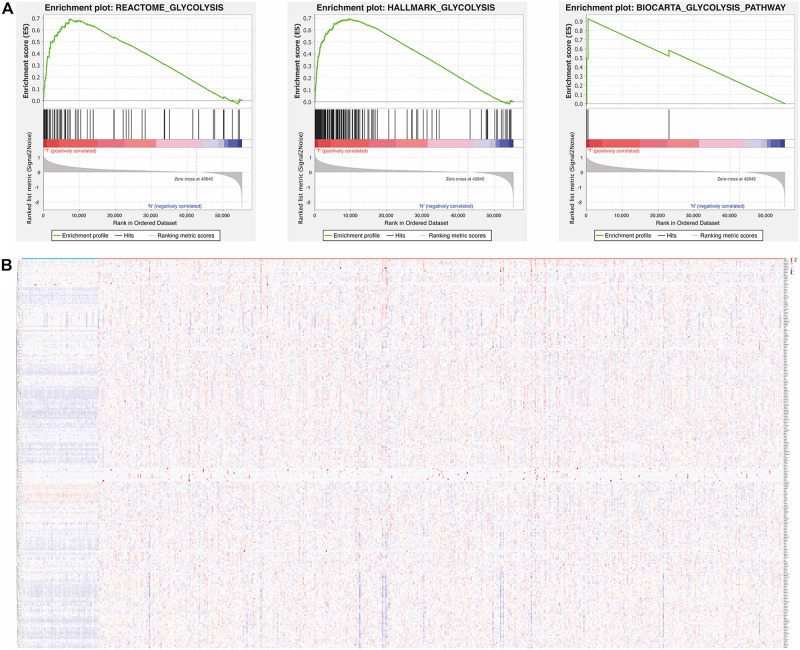
Relationship between glycolysis and tumorigenesis in LUAD. **(A)** Enrichment plots of three gene sets which were differentiated between in LUAD and normal tissues *via* GSEA, *p* < 0.05 was considered to be statistically significant. **(B)** The expression level of 255 glycolysis-related genes in LUAD and normal tissues. Red is up-regulated and blue is down-regulated. Genes are in rows; samples are in columns. LUAD, lung adenocarcinoma; GSEA, gene set enrichment analysis.

**FIGURE 2:**
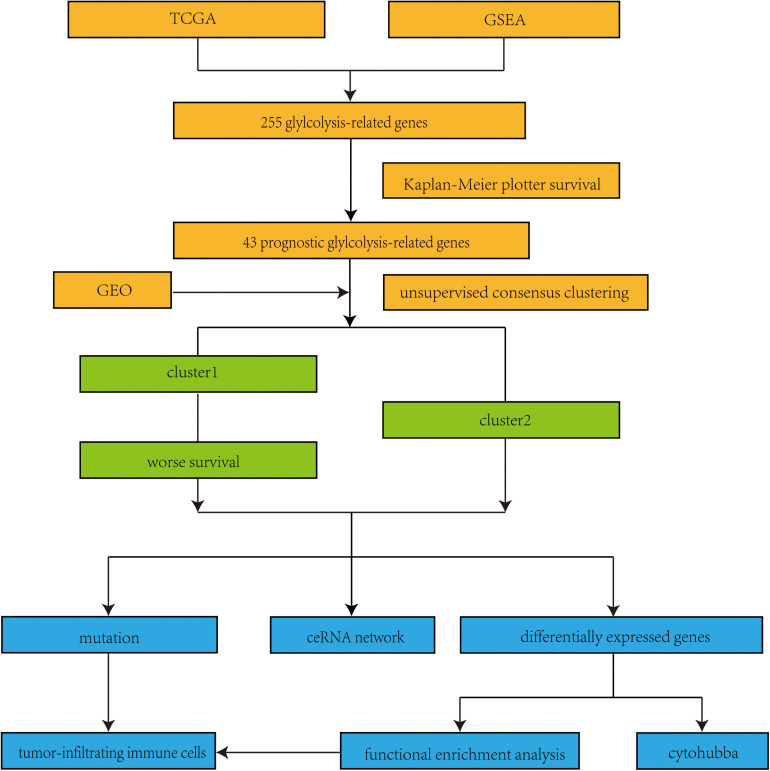
Flow chart of this study. TCGA, the Cancer Genome Atlas; GSEA, Gene set enrichment analysis; GEO, Gene Expression Omnibus; ceRNA, competing endogenous RNA.

We firstly obtained the RNA sequencing data and clinical data of LUAD from TCGA. Then, gene set enrichment analysis (GSEA) was used to further analyze the relationship between glycolysis and LUAD. The GSEA results showed that BIOCARTA GLYCOLYSIS PATHWAY, HALLMARK GLYCOLYSIS, and REACTOME GLYCOLYSIS were significantly enriched in LUAD, which illustrated that glycolysis is involved in the tumorigenesis of LUAD (Figure 1A). According to the GSEA results, 255 glycolysis-related genes were extracted and visualized by a heat map (Figure 1B). The information of 255 glycolysis-related genes is summarized in Supplementary Data Sheet 2. In addition, the flow diagram of this study is illustrated in Figure 2.

### Screening the Prognostic Value of Glycolysis-Related Genes in LUAD

To elucidate the relationship between the glycolysis-related genes and the prognosis of LUAD, we assessed the prognostic values of these 255 glycolysis-related genes using the Kaplan–Meier plotter database and found that only 43 genes were significantly associated with overall survival (all *p* < 0.05) (Table 1) (43 representative figures are shown in Supplementary Figure 1).

**TABLE 1:** Differentially expressed 43 glycolysis-related genes in lung adenocarcinoma.

Gene	logFC	*p*-Value
AK4	2.715034922	7.20E-19
ALDOA	1.305042167	4.72E-27
ARTN	2.852516381	3.05E-20
AURKA	2.774021978	2.56E-31
B4GALT2	1.343368752	4.97E-30
BIK	2.110407599	5.42E-22
CASP6	1.078872032	3.68E-29
CENPA	3.788018525	5.06E-32
CHPF	1.364021632	6.47E-23
CHST4	2.346104416	0.001681461
CLDN3	2.124060254	2.24E-16
CLDN9	2.329920729	1.02E-05
COL5A1	2.156022426	1.99E-21
CTH	1.276548971	1.36E-16
DCN	–1.320135715	4.60E-22
DEPDC1	4.013012326	4.16E-33
DPYSL4	1.386936886	2.34E-07
EFNA3	3.173820767	2.88E-35
EGLN3	3.142581934	2.02E-21
FKBP4	1.263390415	1.29E-22
G6PD	1.459631279	1.89E-05
GAPDH	1.829232597	3.29E-30
GAPDHS	1.828856472	0.00091541
GFPT1	1.299877995	3.08E-30
GMPPA	1.144487386	2.94E-32
GOT1	1.104965663	4.22E-22
GPR87	4.126336521	1.17E-11
HS6ST2	3.800469853	8.00E-27
HSPA5	1.017535778	5.09E-29
IER3	1.104634685	3.94E-10
KIF20A	3.308652489	9.18E-34
LDHA	1.474350133	3.71E-30
MIF	1.324714347	1.23E-20
MIOX	4.340627063	6.58E-24
NDC1	1.17473284	2.58E-26
NUP155	1.482451556	2.84E-30
PFKP	2.274356882	1.22E-26
PGM2L1	2.023614728	6.49E-29
PKP2	1.601542975	1.97E-05
SLC25A10	2.306031843	1.61E-31
SLC25A13	1.218425746	3.39E-29
SPAG4	3.033804993	3.77E-34
TGFA	1.56113819	1.43E-13

### Consensus Clustering of Glycolysis-Related Genes Identified Two Clusters of LUAD

**FIGURE 3:**
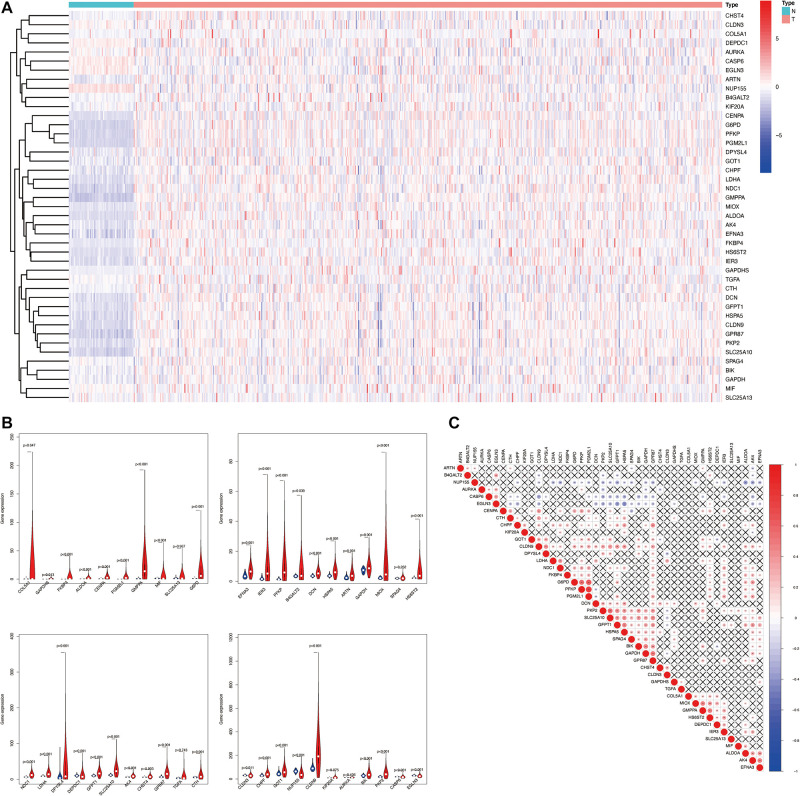
The landscape of 43 glycolysis-related genes in LUAD. **(A)** The expression levels of 43 glycolysis-related genes in LUAD. The red color means tumor tissues and the blue color means the non-tumor tissues. Genes are in rows; samples are in columns. **(B)** Vioplots of 43 glycolysis-related genes in LUAD, *p* < 0.05 was considered to be statistically significant. Blue is normal tissues and red is tumor tissues. **(C)** Spearman correlation analysis of 43 glycolysis-related genes in LUAD. The red color means positive correlation and the blue color means negative correlation. LUAD, lung adenocarcinoma.

**FIGURE 4:**
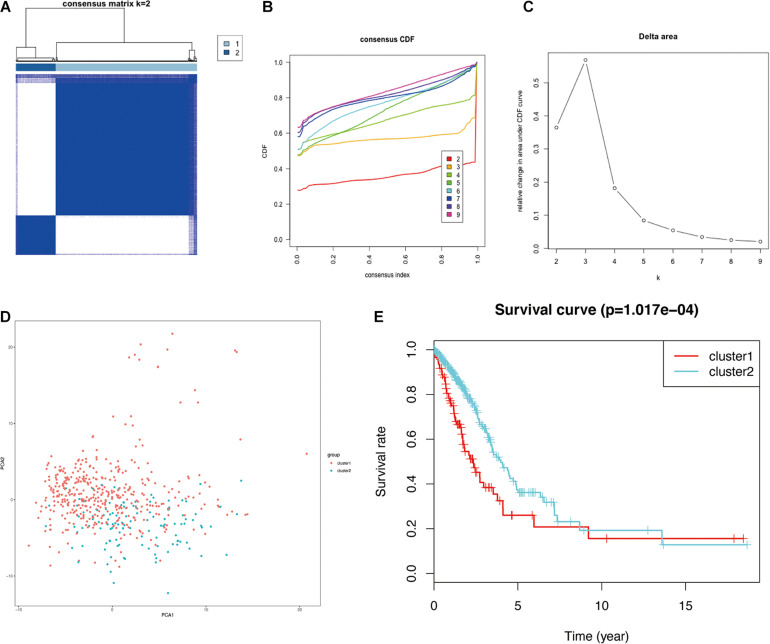
Identification of consensus clusters of glycolysis-related genes in LUAD from the TCGA database. **(A)** Consensus clustering matrix for *k* = 2. **(B)** Consensus clustering CDF for *k* = 2–9. **(C)** Relative change in the area under the CDF curve for *k* = 2–9. **(D)** PCA of the 43 glycolysis-related genes in LUAD. **(E)** Survival curves of each cluster in LUAD. LUAD, lung adenocarcinoma; TCGA, The Cancer Genome Atlas; CDF, cumulative distribution function; PCA, principal component analysis.

Glycolysis plays crucial roles in the biological processes of LUAD, so we next visualized the expression levels and correlation of 43 prognostic glycolysis-related genes (Figure 3). Then, we constructed glycolysis subgroups of LUAD with different biological characters based on 43 glycolysis-related genes. Unsupervised consensus clustering analysis indicated that *k* = 2 was the optimal number of clusters (Figures 4A–C) and principal component analysis (PCA) was used to verify the classification by 43 glycolysis-related genes (Figure 4D). Finally, a total of 534 patients were divided into cluster 1 and cluster 2, and the results showed that cluster 1 was associated with worse overall survival in LUAD patients compared with cluster 2 (*p* < 0.001) (Figure 4E). The specific TCGA patient ID of each cluster is displayed in Supplementary Data Sheet 2.

### Validation of Expression of 43 Glycolysis-Related Genes, Consensus Clustering, and Prognostic Value Using an Independent Cohort

**FIGURE 5:**
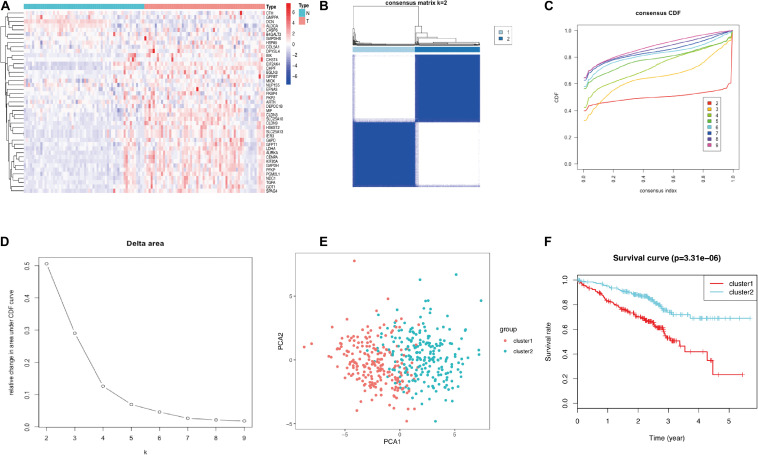
Validation of the gene expression and grouping in GSE72094. **(A)** Heat map of 43 glycolysis-related genes between LUAD and normal tissue. **(B)** Consensus clustering matrix for *k* = 2. **(C)** Consensus clustering CDF for *k* = 2–9. **(D)** Relative change in area under the CDF curve for *k* = 2–9. **(E)** PCA of the 43 glycolysis-related genes in LUAD. **(F)** Survival curves of each cluster in LUAD. LUAD, lung adenocarcinoma; TCGA, The Cancer Genome Atlas; CDF, cumulative distribution function; PCA, principal component analysis.

In order to verify the feasibility of grouping, the gene expression profile of GSE72094 was used for further analyses. The heatmap plot was visualized to further exhibit the distribution of 43 prognostic glycolysis-related genes (Figure 5A). As shown in Supplementary Figure 2, the expression of 42 of 43 prognostic glycolysis-related genes was significantly elevated and one gene was downregulated in LUAD tissues compared with adjacent normal lung tissues in GSE72094 (both *p* < 0.05). Meanwhile, qRT-PCR was also used to verify the expression of these genes. As expected, the results showed that 39 of the 43 genes showed the consistency with the above results, while four genes showed that there is no significance between 16HBE and H1299 (Supplementary Figure 3), which could be caused by the difference between tissue and cell lines. Furthermore, unsupervised consensus clustering analysis and PCA results showed that these two clusters are meaningful and can be verified using GSE72094 (Figures 5B–E). The survival analysis revealed that the joint of glycolysis and gene expression of 43 genes had a significant correlation with the prognosis of LUAD patients (Figure 5F).

### Construction of a 43-Glycolysis-Related Signature

**FIGURE 6:**
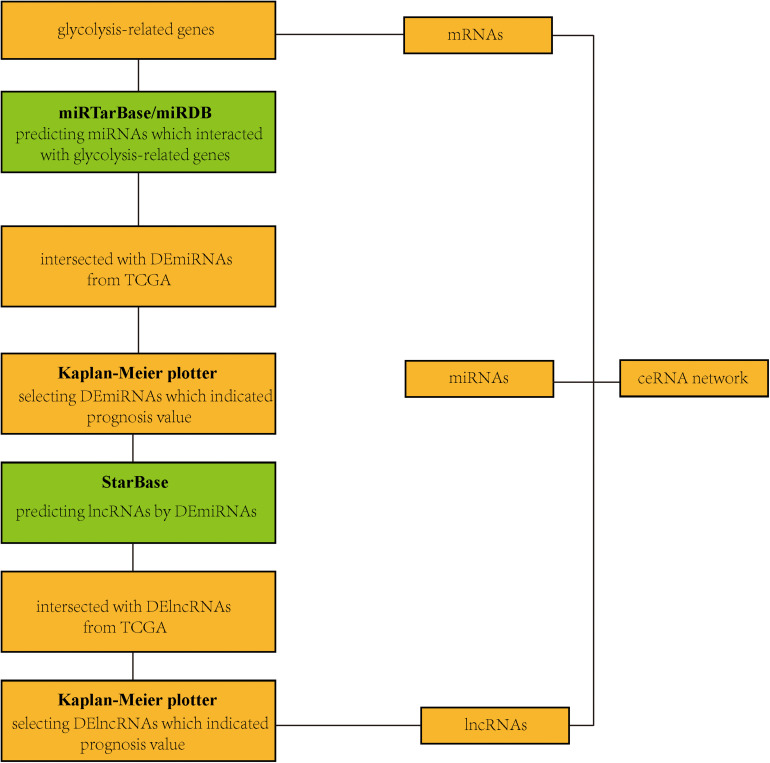
Construction of a ceRNA network. A flow diagram of a 43-glycolysis-related-gene ceRNA network in LUAD. ceRNA, competing endogenous RNA; LUAD, lung adenocarcinoma; DEmiRNAs, differentially expressed miRNAs; TCGA, The Cancer Genome Atlas; DElncRNAs, differentially expressed lncRNAs.

After screening 43 genes, we also constructed a ceRNA network to explore potential upstream mechanisms which may account for the worse overall survival in cluster 1. Using the limma R package according to the standards (*p* < 0.05, | logFC| > 1), a total of 186 DElncRNAs (Supplementary Data Sheet 2) and 150 DEmiRNAs (Supplementary Data Sheet 2) were identified in LUAD. Firstly, the miRNAs targeted by 43 glycolysis-related genes were predicted *via* miRTarBase and miRDB. After overlapping the results, a total of 33 DEmiRNAs (Supplementary Data Sheet 2) were identified from TCGA; the Kaplan–Meier plotter database displayed that 16 (Supplementary Figure 4 and Table 2) of 33 DEmiRNAs were significantly associated with overall survival (all *p* < 0.05). Then, 61 DElncRNAs (Supplementary Data Sheet 2) targeted by 16 DEmiRNAs were predicted *via* StarBase and 13 (Supplementary Figure 5 and Table 3) of 61 DElncRNAs have prognostic values (all *p* < 0.05). Eventually, we constructed a 43-glycolysis-related ceRNA network in LUAD, including 13 DElncRNAs, 16 DEmiRNAs, and 43 glycolysis-related genes (Figure 6).

**TABLE 2:** Differentially expressed miRNA targeted by 43 glycolysis-related genes with prognostic value in lung adenocarcinoma.

miRNA	logFC	*p*-Value
hsa-miR-30c-2-3p	–3.325704214	2.12E-67
hsa-let-7c-5p	–2.194435086	2.46E-40
hsa-miR-140-3p	–1.549497992	7.23E-39
hsa-miR-142-3p	2.498761697	7.28E-33
hsa-let-7b-5p	–1.360489619	1.59E-24
hsa-miR-301a-3p	2.030243634	7.99E-24
hsa-miR-9-5p	3.956683282	2.32E-23
hsa-miR-30a-5p	–1.873152127	2.29E-23
hsa-miR-1-3p	–2.442236467	1.07E-21
hsa-let-7e-5p	–1.446526872	1.01E-19
hsa-miR-218-5p	–1.437453438	2.73E-18
hsa-miR-30d-5p	–1.26709302	1.77E-15
hsa-miR-30b-3p	–1.244337848	8.36E-15
hsa-miR-17-5p	1.08451682	4.84E-14
hsa-miR-331-3p	1.06147245	2.66E-13
hsa-miR-642a-5p	1.453390931	6.31E-11

**TABLE 3:** Differentially expressed lncRNA with prognostic value in lung adenocarcinoma.

lncRNA	logFC	*p*-Value
FENDRR	–4.550706947	1.41E-118
LINC01963	–1.401218255	1.71E-36
LINC02035	–1.129015688	6.75E-34
LINC00511	3.04763667	1.41E-26
C22orf34	–1.357168404	2.94E-26
LINC00261	–2.602384791	2.46E-19
CYTOR	1.2223348	7.67E-15
PWAR6	–1.219476944	8.20E-12
SNHG12	1.046959907	5.49E-11
LINC00665	1.542732963	1.98E-07
FAM30A	1.509315396	8.09E-07
HAGLR	–1.114891174	4.65E-06
MIAT	1.180474479	8.91E-05

### Identification and Functional Enrichment Analysis of the DEGs in Each Cluster

**FIGURE 7:**
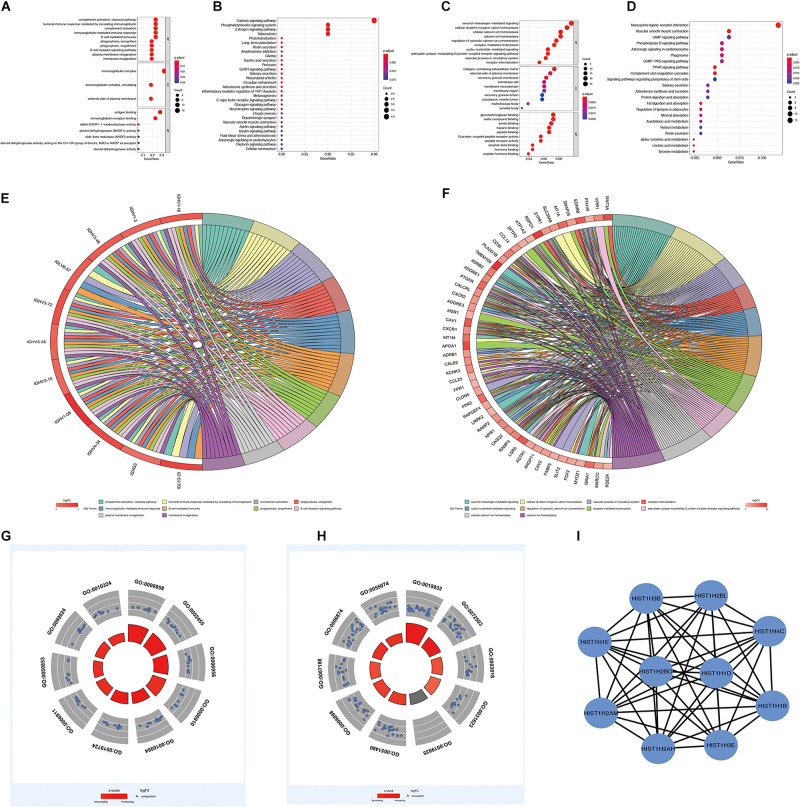
Functional enrichment analysis of DEGs in LUAD. **(A,C)** GO terms of DEGs in cluster 1 and cluster 2. **(B,D)** KEGG pathways enriched for the DEGs in cluster 1 and cluster 2. **(E,F)** The correlation between statistically top 10 DEGs and their GO terms in cluster 1 and cluster 2. **(G,H)** Functional analysis of the genes with higher expression using GO terms in cluster 1 and cluster 2. The outer circle means the expression (logFC) of DEGs in each enriched GO terms: Blue dots indicated the upregulated DEGs. The inner circle means the significance of GO terms (log10-adjusted p values). All *p* < 0.05 was considered to be statistically significant. **(I)** Hub genes selected *via* cytoHubba. GO, Gene Ontology; DEGs, differentially expressed genes; LUAD, lung adenocarcinoma; KEGG, Kyoto Encyclopedia of Genes and Genomes.

To further comprehend the relationship between glycolysis subgroups of LUAD and different patient prognoses, we identified the DEGs in each cluster from TCGA. A total of 384 DEGs (Supplementary Data Sheet 2), including 94 upregulated genes in cluster 1 and 290 upregulated genes in cluster 2 were screened by the limma R package on the basis of *p* < 0.05 and | log2(FC)| > 1. Those upregulated DEGs in cluster 1 are downregulated in cluster 2, which means that the rest of the unmentioned downregulated DEGs in cluster 1 are also elevated in cluster 2. In fact, from different perspectives, the DEGs are also different. For this reason, we only displayed and discussed the upregulated genes in each cluster for their different prognosis. To better realize the function of clustering, we further performed functional enrichment analysis. Upregulated DEGs of cluster 1 were mainly enriched in immune-related biological processes, such as complement activation, classical pathway, humoral immune response mediated by circulating immunoglobulin, and immunoglobulin-mediated immune response (Figure 7A). More specifically, the immune-related functions in cluster 2 mainly include cell chemotaxis, myeloid leukocyte migration, leukocyte chemotaxis, and neutrophil chemotaxis (Figure 7C). The results of KEGG enrichment analysis showed that cluster 1 and cluster 2 were mainly enriched in metabolism-related signaling pathways (Figures 7B,D). Moreover, the functional annotation of DEGs displayed that 10 GO terms were statistically significant (*p* < 0.05) (Figures 7E–H and Tables 4, 5). To identify the biological modules of DEGs in cluster 1, the PPI network was generated by using STRING and visualized by Cytoscape software. The cytoHubba plugin will sort genes by core degree through a variety of algorithms, and genes with high degrees tend to be hub genes. Then, the top 10 hub nodes were identified by the cytoHubba plugin, including HIST1H3B, HIST1H1B, HIST1H1D, HIST1H1E, HIST1H4C, HIST1H2BL, HIST1H2AH, HIST1H2BO, HIST1H3E, and HIST1H2AM (Figure 7I).

**TABLE 4:** The top 10 gene ontology (GO) terms of differentially expressed genes in cluster 1.

Category	ID	Term	Genes	adj_pval
BP	GO:0006958	Complement activation, classical pathway	IGHV1-18, IGHV1-3, IGHV3-48, IGLV6-57, IGHV3-72, IGHV3-35, IGHV3-15, IGHV1-58, IGHV4-34, IGHG3, IGLV3-25	2.53E-11
BP	GO:0002455	Humoral immune response mediated by circulating immunoglobulin	IGHV1-18, IGHV1-3, IGHV3-48, IGLV6-57, IGHV3-72, IGHV3-35, IGHV3-15, IGHV1-58, IGHV4-34, IGHG3, IGLV3-25	3.63E-11
BP	GO:0006956	Complement activation	IGHV1-18, IGHV1-3, IGHV3-48, IGLV6-57, IGHV3-72, IGHV3-35, IGHV3-15, IGHV1-58, IGHV4-34, IGHG3, IGLV3-25	1.12E-10
BP	GO:0006910	Phagocytosis, recognition	IGHV1-18, IGHV1-3, IGHV3-48, IGHV3-72, IGHV3-35, IGHV3-15, IGHV1-58, IGHV4-34, IGHG3	1.35E-10
BP	GO:0016064	Immunoglobulin-mediated immune response	IGHV1-18, IGHV1-3, IGHV3-48, IGLV6-57, IGHV3-72, IGHV3-35, IGHV3-15, IGHV1-58, IGHV4-34, IGHG3, IGLV3-25	9.18E-10
BP	GO:0019724	B cell-mediated immunity	IGHV1-18, IGHV1-3, IGHV3-48, IGLV6-57, IGHV3-72, IGHV3-35, IGHV3-15, IGHV1-58, IGHV4-34, IGHG3, IGLV3-25	9.18E-10
BP	GO:0006911	Phagocytosis, engulfment	IGHV1-18, IGHV1-3, IGHV3-48, IGHV3-72, IGHV3-35, IGHV3-15, IGHV1-58, IGHV4-34, IGHG3	2.08E-09
BP	GO:0050853	B cell receptor signaling pathway	IGHV1-18, IGHV1-3, IGHV3-48, IGHV3-72, IGHV3-35, IGHV3-15, IGHV1-58, IGHV4-34, IGHG3	3.02E-09
BP	GO:0099024	Plasma membrane invagination	IGHV1-18, IGHV1-3, IGHV3-48, IGHV3-72, IGHV3-35, IGHV3-15, IGHV1-58, IGHV4-34, IGHG3	3.02E-09
BP	GO:0010324	Membrane invagination	IGHV1-18, IGHV1-3, IGHV3-48, IGHV3-72, IGHV3-35, IGHV3-15, IGHV1-58, IGHV4-34, IGHG3	4.57E-09

*Note. BP, biological process.*

**TABLE 5:** The top 10 gene ontology (GO) terms of differentially expressed genes in cluster 2.

Category	ID	Term	Genes	adj_pval
BP	GO:0019932	Second-messenger-mediated signaling	PTH1R, EDNRB, S1PR1, ATP1A2, CD36, TMEM100, ADRB2, ADGRE1, PTGFR, CALCRL, CXCR2, ADGRE3, CXCR1, MT1M, ADRB1, FPR1, FPR2, RAPGEF4, LRRK2, RAMP2, NPR1, CASQ2, RAMP3, LGR5, AGTR1, MYOZ1, PDE2A	6.75E-08
BP	GO:0072503	Cellular divalent inorganic cation homeostasis	PTH1R, EDNRB, MT1A, SLC39A8, S1PR1, ATP1A2, CCL14, CD36, PLA2G1B, PTGFR, CXCR2, CAV1, CXCR1, MT1M, CALB2, KCNK3, CCL23, FPR1, FPR2, CASQ2, RAMP3, AGTR1, CAV2, FGF2, GRIA1	4.66E-06
BP	GO:0003018	Vascular process in circulatory system	EDNRB, ATP1A2, CD36, ADRB2, CXCR2, CAV1, ADRB1, CLDN5, RAMP2, NPR1, AGTR1, ANGPT1, FABP5, SLIT2, PDE2A	1.30E-05
BP	GO:0031623	Receptor internalization	WNT3A, SNAP25, RSPO1, CD36, CALCRL, CXCR2, CAV1, CXCR1, RAMP2, RAMP3, ANGPT1, GRIA1	1.30E-05
BP	GO:0019935	Cyclic-nucleotide-mediated signaling	PTH1R, EDNRB, S1PR1, CD36, ADRB2, ADGRE1, PTGFR, CALCRL, ADGRE3, ADRB1, RAPGEF4, RAMP2, NPR1, RAMP3, LGR5, PDE2A	1.30E-05
BP	GO:0051480	Regulation of cytosolic calcium ion concentration	PTH1R, EDNRB, S1PR1, ATP1A2, CD36, PLA2G1B, PTGFR, CXCR2, CAV1, CXCR1, CALB2, KCNK3, FPR1, FPR2, CASQ2, RAMP3, AGTR1, CAV2, FGF2, GRIA1	1.30E-05
BP	GO:0006898	Receptor-mediated endocytosis	WNT3A, SNAP25, RSPO1, SFTPD, CD36, ADRB2, CALCRL, CXCR2, MSR1, CAV1, CXCR1, APOA1, FPR2, RAMP2, RAMP3, ANGPT1, CAV2, GRIA1, MARCO	1.49E-05
BP	GO:0007188	Adenylate cyclase-modulating G protein-coupled receptor signaling pathway	VIPR1, PTH1R, S1PR1, ADRB2, ADGRE1, PTGFR, CALCRL, ADGRE3, ADRB1, FPR1, FPR2, RAMP2, RAMP3, LGR5, MARCO, PDE2A	1.61E-05
BP	GO:0006874	Cellular calcium ion homeostasis	PTH1R, EDNRB, S1PR1, ATP1A2, CCL14, CD36, PLA2G1B, PTGFR, CXCR2, CAV1, CXCR1, CALB2, KCNK3, CCL23, FPR1, FPR2, CASQ2, RAMP3, AGTR1, CAV2, FGF2, GRIA1	2.44E-05
BP	GO:0055074	Calcium ion homeostasis	PTH1R, EDNRB, S1PR1, ATP1A2, CCL14, CD36, PLA2G1B, PTGFR, CXCR2, CAV1, CXCR1, CALB2, KCNK3, CCL23, FPR1, FPR2, CASQ2, RAMP3, AGTR1, CAV2, FGF2, GRIA1	3.45E-05

*BP, biological process.*

### Identification of the Mutation Profile Features From Each Cluster

**FIGURE 8:**
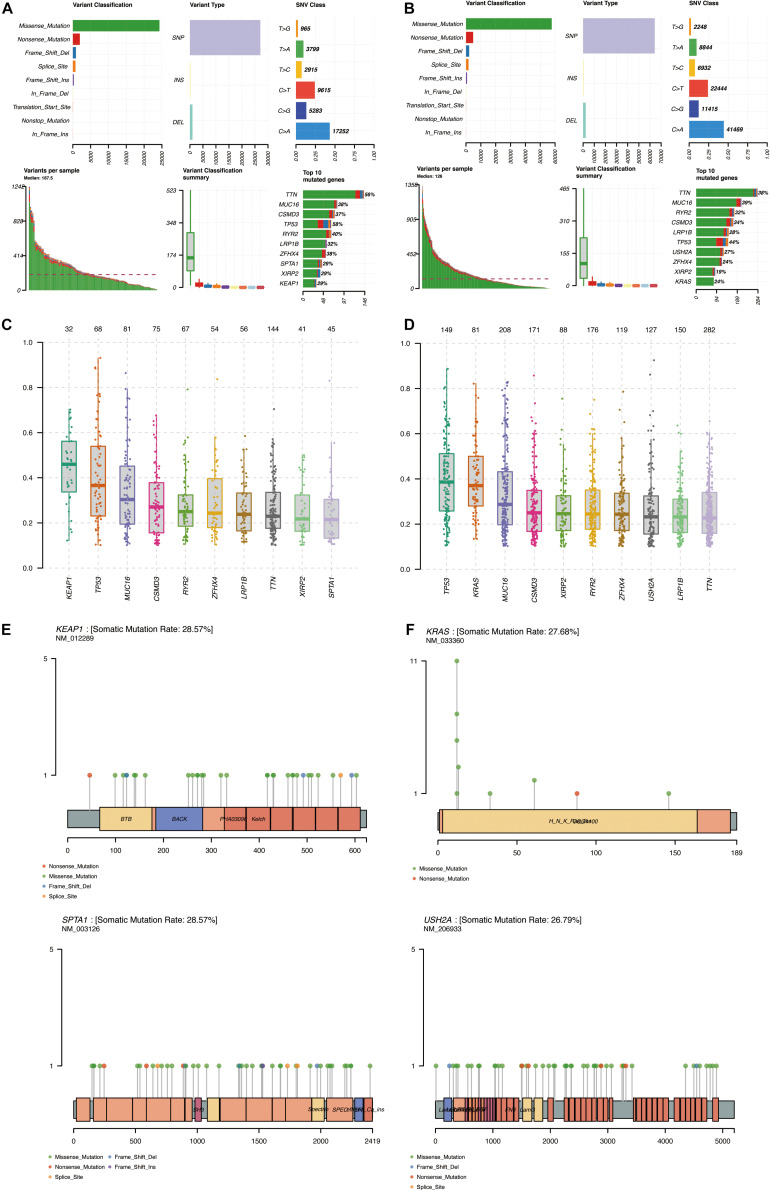
Summary of mutation profiling of LUAD in the MAF file. **(A,B)** Statistical calculations of mutation types in cluster 1 and cluster 2. **(C,D)** VAF of LUAD in cluster 1 and cluster 2. **(E,F)** Lollipop plot of special mutated genes in cluster 1 and cluster 2. MAF, minor allele frequency; LUAD, lung adenocarcinoma; VAF, variant allele frequency.

**FIGURE 9:**
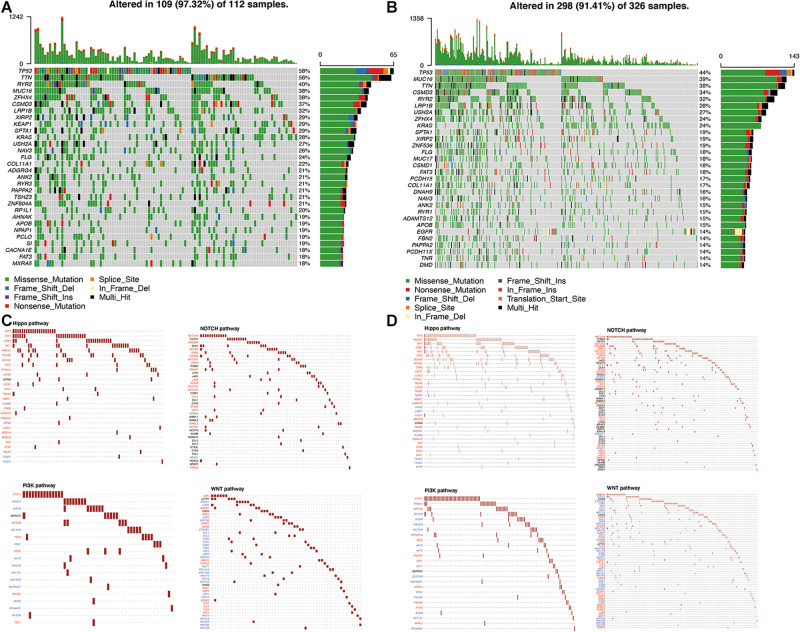
Visualization of the mutation profile signature in each cluster. **(A,B)** Waterfall plot of mutation profiles in cluster 1 and cluster 2 in LUAD samples. The comments below mean the mutation types. The bar chart on the right shows the distribution of mutant types in the top 30 genes. **(C,D)** The oncogenic pathways of mutant genes in cluster 1 and cluster 2. TMB, tumor mutation burden; LUAD, lung adenocarcinoma.

The simple nucleotide variation data of 561 LUAD samples were downloaded from TCGA and processed by VarScan software. The landscape of mutation data was visualized using the “maftools” package. Mutation profile features indicated that missense mutation was the most common type in Variant Classification, single-nucleotide polymorphism formed the nucleus of variant type, and C>A accounted for more components than other single-nucleotide variants in cluster 1 and cluster 2. The top 10 mutated signatures were visualized by a horizontal histogram, and the results showed that the mutated SPTA1 (29%) and KEAP1 (29%) are unique in cluster 1 among the top 10 mutated genes, as well as USH2A (27%) and KRAS (24%) in cluster 2, which may be the underlying mechanism of grouping (Figures 8A,B). Besides, the variant allele frequency (VAF) of LUAD is visualized in Figures 8C,D and a lollipop plot displayed these unique mutation points on the protein structure (Figures 8E,F). A waterfall plot displayed the mutation information in each sample, and in cluster 1, seven genes were mutated by >30%: TP53 (58%), TTN (56%), RYR2 (40%), MUC16 (38%), ZFHX4 (38%), CSMD3 (37%), and LRP1B (32%). In cluster 2, five genes were mutated by >30%: TP53 (44%), MUC16 (39%), TTN (38%), CSMD3 (34%), and RYR2 (32%) (Figures 9A,B). Besides, mutated oncogenic pathways were also visualized in each cluster (Figures 9C,D).

### Identification of Immune Cell Infiltration Signatures of Each Cluster

**FIGURE 10:**
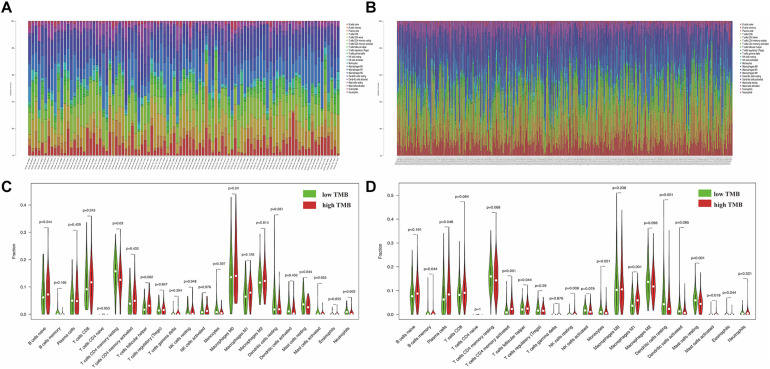
Illustration of immune cell infiltrates features between low-TMB and high-TMB groups in each cluster. **(A,B)** The percent of 22 types of tumor-infiltrating immune cell in cluster 1 and cluster 2. **(C,D)** The difference of 22 types of immune cells between high and low TMB groups in cluster 1 and cluster 2. The higher TMB and lower TMB were shown in red and green. TMB, tumor mutation burden.

The functional enrichment analysis showed that DEGs were involved in cell chemotaxis and immune-related biological processes, which lead us further to analyze the correlation of TMB with immune signatures in each cluster. Based on the CIBERSORT algorithm, we revealed the proportions of different immune cells in specific patients in each cluster. The percentage of 22 types of tumor-infiltrating immune cell in each cluster was visualized (Figures 10A,B). Subsequently, we indicated the differential abundance of immune cells in the low-TMB and high-TMB groups *via* violin plots. Memory resting CD4+ T cells (*p* = 0.03), resting mast cells (*p* = 0.044), and neutrophils (*p* = 0.002) showed higher infiltrating levels in the low-TMB group in cluster 1 (Figure 10C). Moreover, the infiltration levels of memory B cells (*p* = 0.044), plasma cells (*p* = 0.048), activated memory CD4+ T cells (*p* < 0.001), resting NK cells (*p* = 0.008), M1 macrophages (*p* < 0.001), and activated mast cells (*p* = 0.019) were higher in the high-TMB group in cluster 2, However, monocytes (*p* = 0.021), resting dendritic cells (*p* < 0.001), and resting mast cells (*p* < 0.001) showed a higher infiltrating level in the low-TMB group (Figure 10D).

## Discussion

Lung adenocarcinoma is one of the prevalent malignant tumors with increasing incidence, mortality, and poor prognosis (Nakamura and Saji, 2014; Hirsch et al., 2017). Meanwhile, LUAD displayed unique metabolic characteristics with high glycolytic activity, which is one of the most crucial hallmarks of cancer (Hanahan and Weinberg, 2011). It is reported that PPP1R14B-AS1 overexpression was associated with poor prognosis in LUAD (Yang et al., 2020). Inhibition of glycolysis could regulate the cell survival of LUAD (Farah et al., 2012). However, the relationship between glycolysis-related gene signatures and LUAD prognosis is still unclear so far. In this study, 43 differentially expressed glycolysis-related genes were related to the overall survival for LUAD patients. An appropriate recognition of tumor subgroups is necessary as it may affect patients’ prognosis and consideration of molecular detection. On the basis of 43 prognostic genes, we classified two subgroups of LUAD (cluster 1 and 2) by applying unsupervised consensus clustering, which had different clinical prognosis values. Therefore, we analyzed the possible mechanisms of different prognosis from multiple perspectives.

A subgroup-specific molecular study transforms the biological characteristics of LUAD into clinical prognostic subgroups to find potential biomarkers. Patients with different subgroups of LUAD had significantly different survival prognoses. In the present study, survival analysis results showed that cluster 1 was associated with worse overall survival for LUAD patients (*p* < 0.001). To explore the underlying upstream molecular mechanism which may account for the worse overall survival in cluster 1, we constructed a gene signature related to glycolysis, including 13 DElncRNAs, 16 DEmiRNAs, and 43 glycolysis-related genes and then verified the prognostic value of this gene signature in LUAD (all *p* < 0.05). In addition, the difference of gene expression level, immune infiltration signatures, and gene mutation infiltration signatures, and gene mutation information in cluster 1 was compared with that in cluster 2. The transformation of metabolic pathways is usually regulated by specific gene expression, and genetic signatures composed of multiple genes have been developed to predict the prognostic value of clinical cancer patients. According to the different clusters, we screened that 94 DEGs were enriched in immune-related biological processes in cluster 1. The top 10 hub genes were selected to help identify subgroups, and we found that histone modifications, one of epigenetic modifications, participated in the occurrence of cancer, which also could be regarded as a prognostic biomarker (Dworkin et al., 2009). Among these top 10 hub genes, HIST1H3B/C-K27M-mutated tumors exhibit a pro-angiogenic/hypoxic signature in diffuse intrinsic pontine gliomas (Castel et al., 2015). Copy number variations of HIST1H1B were connected with cellular development and proliferation in melanoma (Fidalgo et al., 2016). The expression of HIST1H2AH was higher in esophageal squamous cell carcinoma tissues than in adjacent non-tumorous tissues (Wang et al., 2019). Ten histone-coding genes here might regulate tumorigenesis to affect prognosis in cluster 1 for LUAD patients.

Compared with a single prediction model, the effect of analysis results combined with multiple perspectives can provide a more accurate prediction. Mutation was regarded as a therapy target to improve the prognosis of multiply tumors. Previous researches have reported that targeting SGLT2 may intercept LUAD progression at early stages (Scafoglio et al., 2018). SETD2-mutated LUAD patients exhibited poor recurrence-free survival (Kadara et al., 2017). In this study, we found that the mutated SPTA1 (29%) and KEAP1 (29%) are unique in cluster 1 among the top 10 mutated genes, as well as USH2A (27%) and KRAS (24%) in cluster 2. Furthermore, dysregulation of signaling pathways can also change in cancer metabolism to affect patients’ prognosis (Huang et al., 2016). For example, inactivation of the Hippo pathway is connected with the occurrence of various tumors (Panciera et al., 2017). Notch can promote glycolytic metabolism in T cell acute lymphoblastic leukemia (Palomero et al., 2007). Overexpressed SKA3 correlates with poor prognosis through the EGFR-PI3K-Akt axis in LUAD (Hu et al., 2020). Overexpression of CHAP2 may inhibit LUAD cell proliferation and correlate with high survival rate *via* the WNT signal pathway (Shang et al., 2019). In this study, the enrichment pathways of mutant genes are mainly WNT, PI3K, NOTCH, and Hippo signaling pathways in each cluster, which demonstrated that mutated genes contribute to different prognostic effects of LUAD through these pathways. However, the detailed mechanisms need further investigation.

The highly acidic microenvironment produced by tumor glycolysis may affect the infiltration of immune cells to varying degrees, eventually leading to immune escape and tumor progression (Gill et al., 2016; Cascone et al., 2018). Meanwhile, immune cell infiltration has a double feature to affect tumor progression, not only inhibiting the occurrence of tumor but also playing a pro-tumor role (Terlizzi et al., 2014). It is reported that lymphocyte infiltration has been connected with improved survival in NSCLC (Zeng et al., 2016). Mutated genes may generate neoantigens that increase lymphocyte infiltration in the tumor microenvironment. In this study, we found that the high TMB group has higher fractions of memory B cells (*p* = 0.044), plasma cells (*p* = 0.048), activated memory CD4+ T cells (*p* < 0.001), resting NK cells (*p* = 0.008), M1 macrophages (*p* < 0.001), and activated mast cells (*p* = 0.019) in cluster 2, which indicated that glycolysis-related cluster 2 with high TMB contributed to an immunomodulatory tumor microenvironment. These results indicated that TMB could affect the immune cell infiltration signatures and high TMB tends to cause the chemotaxis of immune cells in LUAD.

## Conclusion

In conclusion, this study is the first to report a 43-gene prognostic signature related to glycolysis in LUAD. Furthermore, we constructed a complicated signature, among which the combination analysis of TMB and tumor-infiltrating immune cells was used to assess prognosis in different tumor subgroups of LUAD. Our study may provide a novel sight to realize the mechanisms of glycolysis and identify original gene targets for LUAD patients in the future.

## Data Availability Statement

The original contributions presented in the study are included in the article/Supplementary Material, further inquiries can be directed to the corresponding author.

## Author Contributions

CH designed the research, analyzed the data, and wrote the manuscript. M-YZ and RL analyzed the data. T-TL and J-PL performed the data analysis and interpretation of the data. Y-QQ assisted with the study design and revised the manuscript. All authors contributed to the article and approved the submitted version.

## Conflict of Interest

The authors declare that the research was conducted in the absence of any commercial or financial relationships that could be construed as a potential conflict of interest.

## Footnotes

1 https://portal.gdc.cancer.gov/

2 http://www.ncbi.nlm.nih.gov/geo/

3 http://www.broadinstitute.org/gsea/index.jsp

4 http://software.broadinstitute.org/gsea/msigdb/

5 http://mirtarbase.mbc.nctu.edu.tw/

6 http://www.mirdb.org/

7 http://starbase.sysu.edu.cn/

8 https://kmplot.com/analysis/
