# Supplementary material for: Glycolysis Define Two Prognostic Subgroups of Lung Adenocarcinoma With Different Mutation Characteristics and Immune Infiltration Signatures
Source: Front Cell Dev Biol. 2021 Jul 22;9:645482. doi: 10.3389/fcell.2021.645482 (PMC8339438; doi:10.3389/fcell.2021.645482)
Supplement: Supplementary file 1 [file Data_Sheet_1.docx]

**Supplementary figure legends**

**Supplementary Figure S1**. Kaplan–Meier curve analysis of 43 glycolysis-related genes in lung adenocarcinoma.

**Supplementary Figure S2**. Validation of 43 glycolysis-related genes expression in GEO dataset.

**Supplementary Figure S3**. The expression of 43 glycolysis-related genes using qRT-PCR. The data are expressed as the mean of four independent experiments ± standard deviation, *P < 0.05, **P < 0.01 and ***P < 0.001 vs. the normal lung group.

**Supplementary Figure S4**. Kaplan–Meier curve analysis of 16 differentially expressed miRNAs in lung adenocarcinoma.

**Supplementary Figure S5**. Kaplan–Meier curve analysis of 13 differentially expressed lncRNAs in lung adenocarcinoma.
