# Supplementary figures and images for: Glycolysis Define Two Prognostic Subgroups of Lung Adenocarcinoma With Different Mutation Characteristics and Immune Infiltration Signatures
Source: Front Cell Dev Biol. 2021 Jul 22;9:645482. doi: 10.3389/fcell.2021.645482 (PMC8339438; doi:10.3389/fcell.2021.645482)

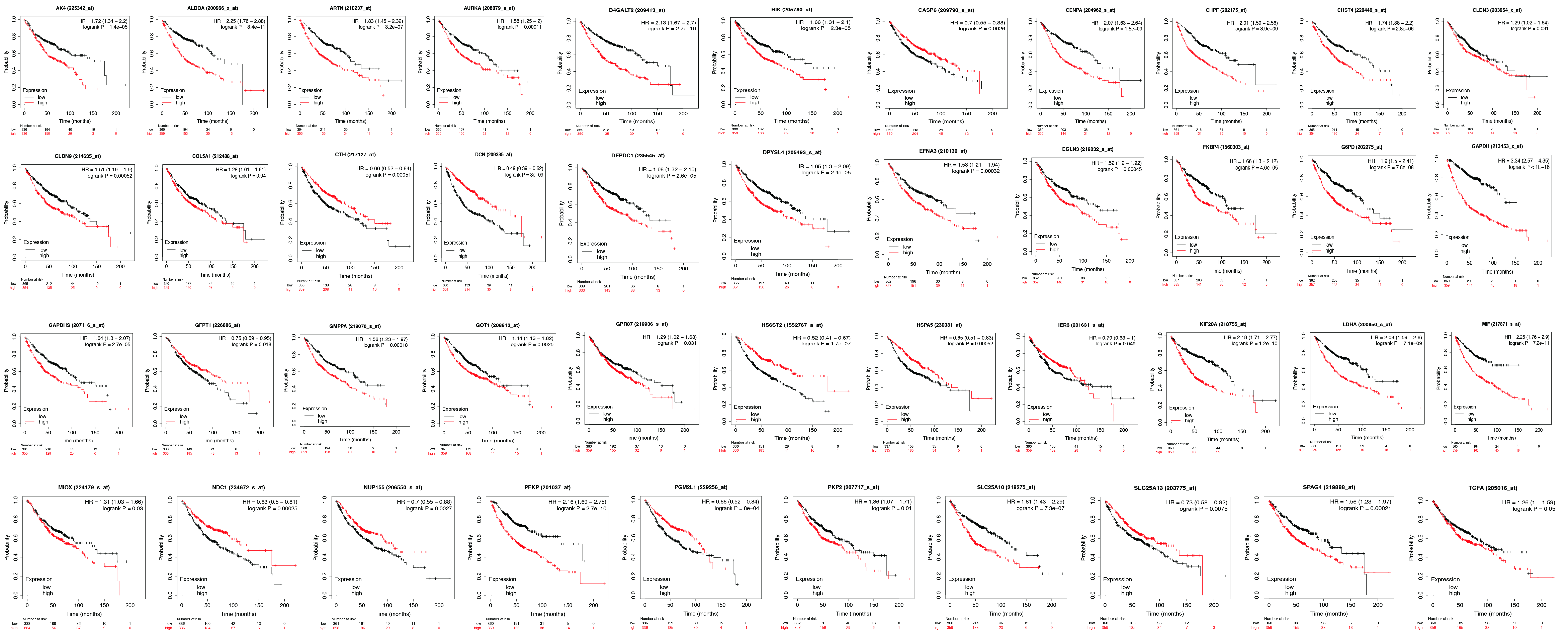

Supplement: Supplementary file 3 [file Image_1.TIF]

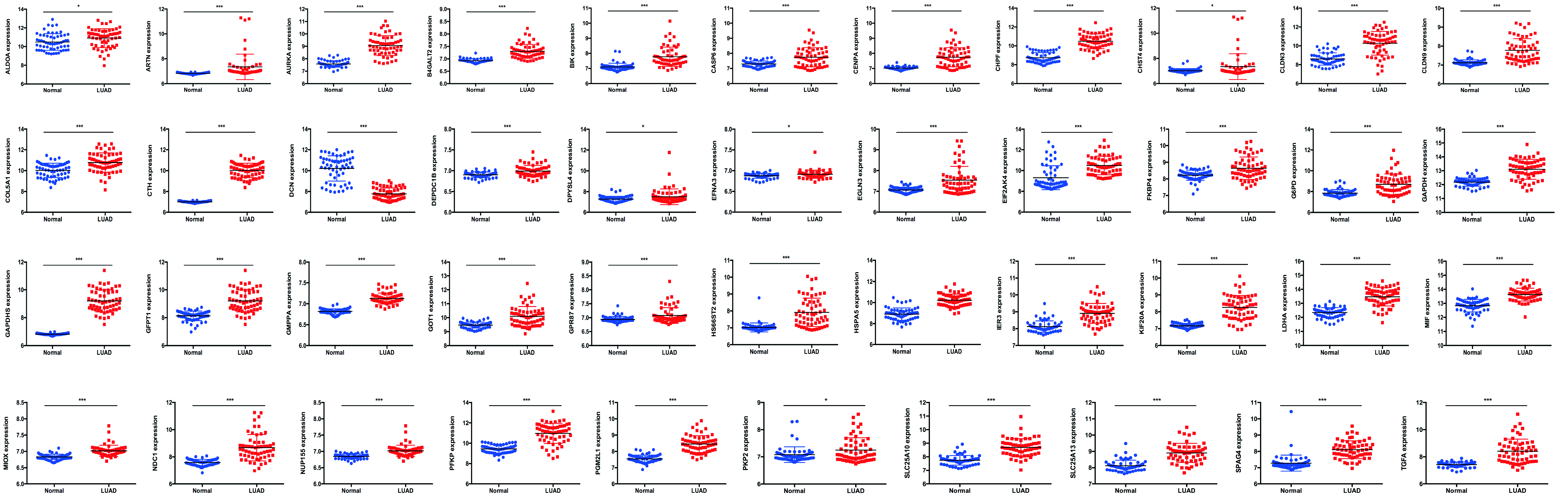

Supplement: Supplementary file 4 [file Image_2.TIF]

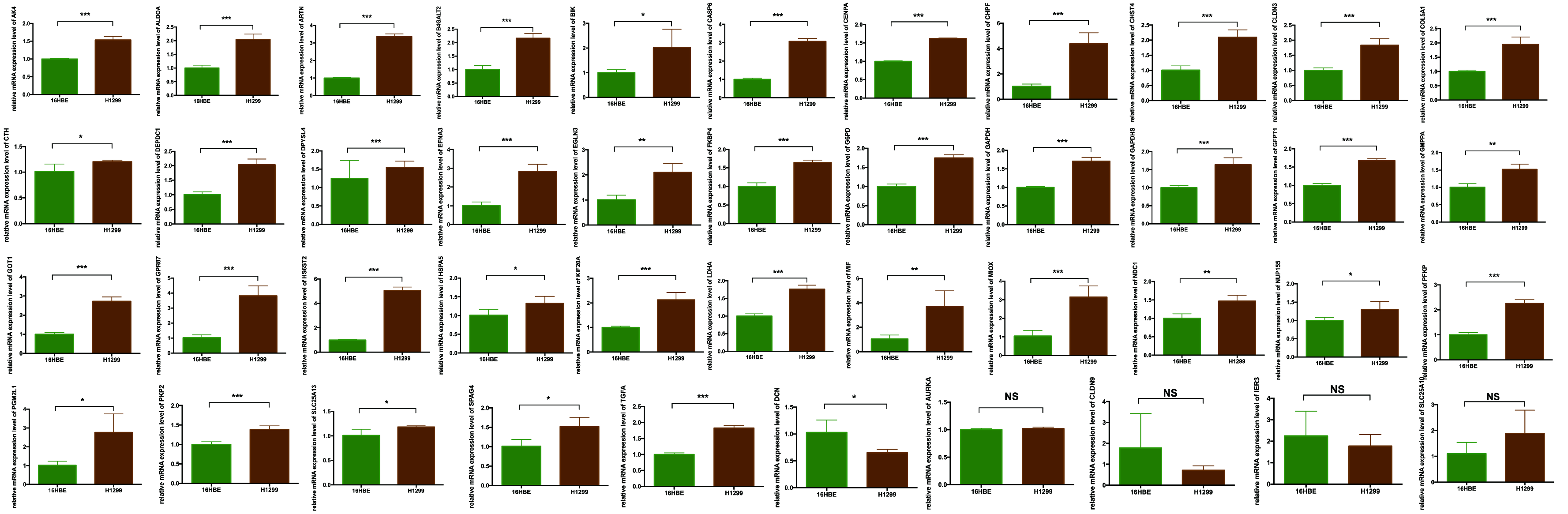

Supplement: Supplementary file 5 [file Image_3.TIF]

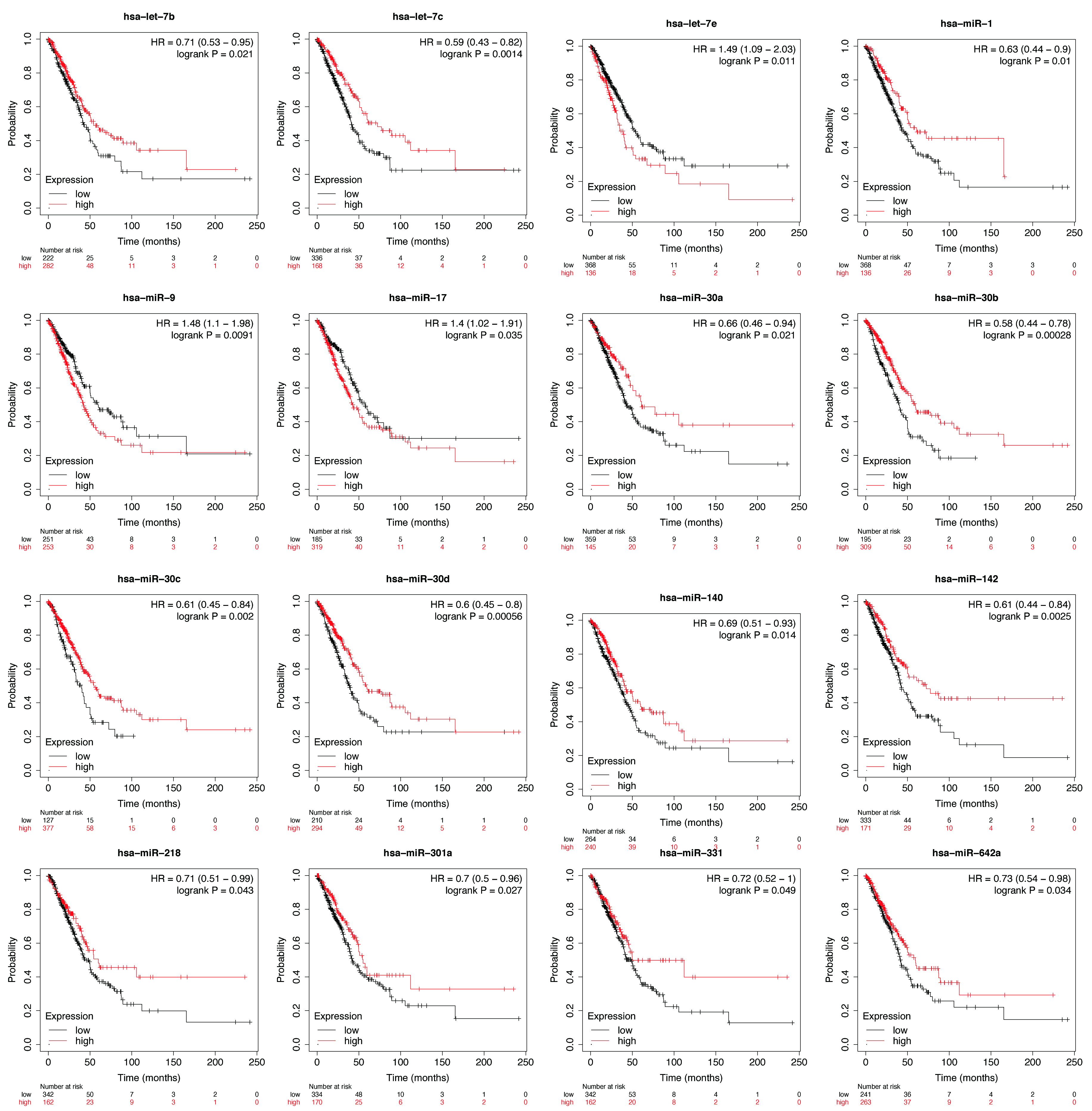

Supplement: Supplementary file 6 [file Image_4.TIF]

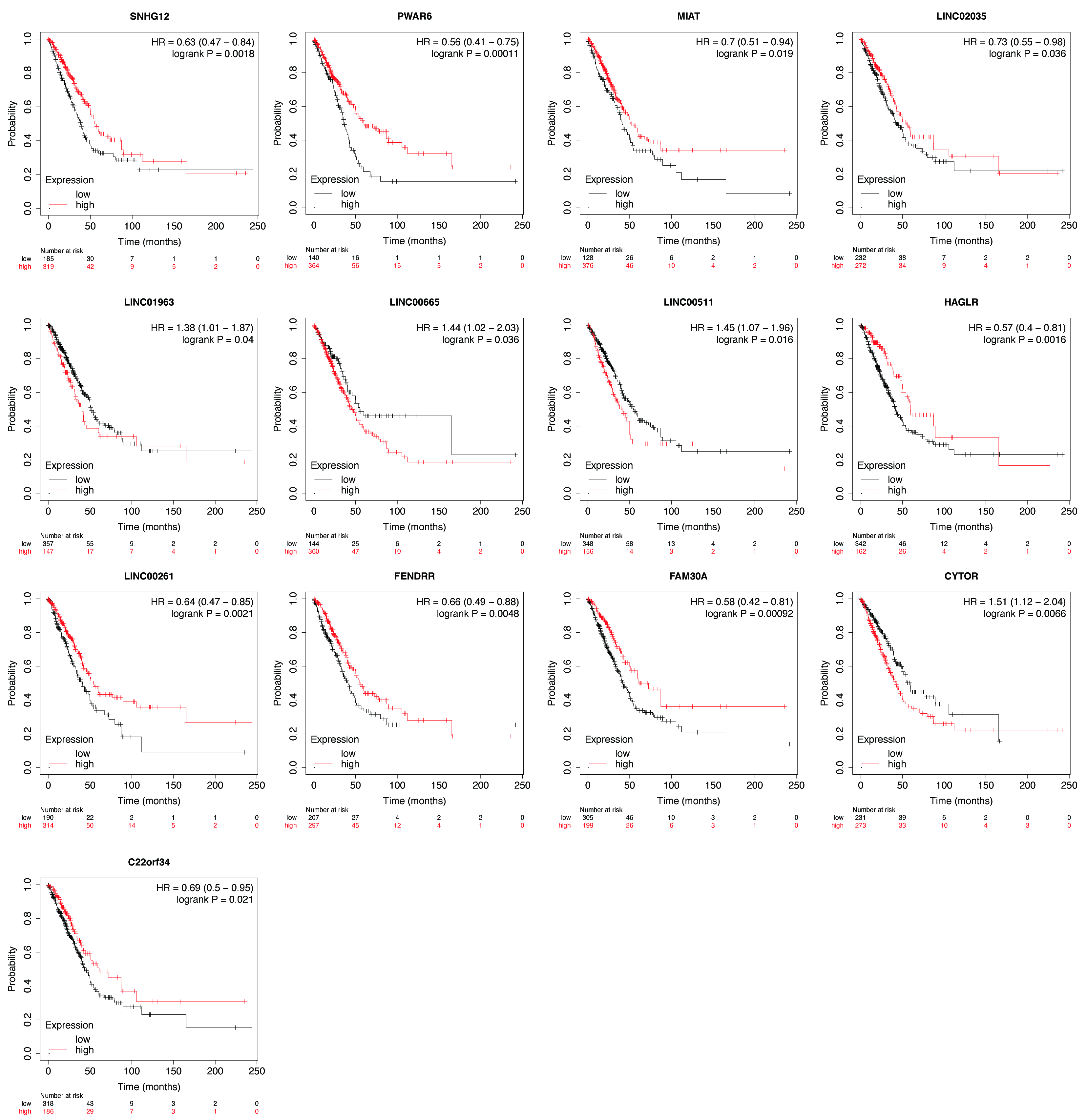

Supplement: Supplementary file 7 [file Image_5.TIF]
